# THαβ Immunological Pathway as Protective Immune Response against Prion Diseases: An Insight for Prion Infection Therapy

**DOI:** 10.3390/v14020408

**Published:** 2022-02-17

**Authors:** Adam Tsou, Po-Jui Chen, Kuo-Wang Tsai, Wan-Chung Hu, Kuo-Cheng Lu

**Affiliations:** 1 Department of Neurology, Taipei Tzu Chi Hospital, Buddhist Tzu Chi Medical Foundation, New Taipei City 231, Taiwan; adam@livemail.tw; 2 Department of Pediatrics, Taoyuan Armed Forces General Hospital, Taoyuan City 325, Taiwan; mythnobody0221@gmail.com; 3 Department of Medical Research, Taipei Tzu Chi Hospital, Buddhist Tzu Chi Medical Foundation, New Taipei City 231, Taiwan; kwtsai6733@gmail.com (K.-W.T.); kuochenglu@gmail.com (K.-C.L.); 4 Department of Clinical Pathology and Medical Research, Taipei Tzu Chi Hospital, Buddhist Tzu Chi Medical Foundation, New Taipei City 231, Taiwan; 5 Division of Nephrology, Department of Medicine, Fu-Jen Catholic University Hospital, School of Medicine, Fu-Jen Catholic University, New Taipei City 243, Taiwan

**Keywords:** prion, immunity, interleukin-10, type 1 interferons, microglia

## Abstract

Prion diseases, including Creutzfeldt–Jakob disease, are mediated by transmissible proteinaceous pathogens. Pathological changes indicative of neuro-degeneration have been observed in the brains of affected patients. Simultaneously, microglial activation, along with the upregulation of pro-inflammatory cytokines, including IL-1 or TNF-α, have also been observed in brain tissue of these patients. Consequently, pro-inflammatory cytokines are thought to be involved in the pathogenesis of these diseases. Accelerated prion infections have been seen in interleukin-10 knockout mice, and type 1 interferons have been found to be protective against these diseases. Since interleukin-10 and type 1 interferons are key mediators of the antiviral THαβ immunological pathway, protective host immunity against prion diseases may be regulated via THαβ immunity. Currently no effective treatment strategies exist for prion disease; however, drugs that target the regulation of IL-10, IFN-alpha, or IFN-β, and consequently modulate the THαβ immunological pathway, may prove to be effective therapeutic options.

## 1. Introduction

Prion diseases, a debilitating example of which is Creutzfeldt–Jakob disease (CJD), are caused by transmissible proteinaceous pathogens. Patients with prion disease show degenerative changes in the brain and nervous tissues that are progressive and eventually fatal. Currently, no effective medications exist for the treatment of these detrimental diseases, and the pathogenesis and immunological responses associated with prion diseases remain unclear. This review discusses the host immunological pathways that attempt to limit prion diseases.

Prions are essentially defined by the protein-only hypothesis, which states that these pathogens comprise only proteins and lack any genetically inherited nucleic acid material. Their discovery led to the abandonment of the scientific dogma that only DNA- or RNA-containing organisms could be transmitted in infectious diseases. This was further established by the discovery of only proteinaceous content, and the lack of either DNA or RNA, from scrapie-infected mouse and hamster brains. Additionally, this proteinaceous content was found to be transmissible and infectious. Prions consist of scrapie prion protein (PrP^sc^), which is a conformer of cellular prion protein (PrP^c^). PrP^sc^ aggregates recruit PrP^c^, which results in template-mediated misfolding to cause conformational change of PrP^c^ to PrP^sc^. The pivotal research that led to the discovery of these pathogens earned Dr. Prusiner the Nobel Prize for Physiology and Medicine in 1997 [1,2,3].

Pathological findings in prion diseases usually manifest as spongiform formations within nervous tissues. These pathogenic proteins infect the central nervous system (CNS) tissues and, subsequently, induce PrP^c^ to transform and acquire their structure, thereby enabling their transmission within CNS tissue. The prion transmission cycle, involving PrP^c^ that are anchored in the cellular membranes of neuronal tissue, results in neurotoxicity that is characterized by typical pathological spongiform changes in infected brain tissue. The host immunological pathways involved in prion diseases are unclear, and neuro-immunological phenomena may play crucial roles in the pathophysiology of prion infections. Consequently, immune modulation may be exploited as a therapeutic strategy for the treatment of these diseases. This review aims to discuss the clinical presentation, immune pathogenesis, and possible therapeutic strategies for the treatment of prion diseases [4].

## 2. Sporadic Human Prion Disease

### 2.1. Sporadic Creutzfeldt–Jakob Disease (sCJD)

The clinical features of CJD include rapid, progressive dementia accompanied by ataxia, myoclonus, visual abnormalities, and other manifestations of nervous system dysfunction including typical periodic sharp wave complexes in electroencephalography (EEG). Neuropathological observations include abnormal prion protein aggregation, spongiform changes, neuronal loss, and gliosis. Despite these common characteristics, the disease has been known to display great phenotypic variability ever since it was first described [5]. Regarding the combination of the methionine (M) valine (V) polymorphism at codon 129 and the prion PrP^sc^ confirmation type (type 1 or type 2), sporadic CJD can be classified into subtypes, including VV1, VV2, MM1, MM2, MV1, and MV2. The VV2 subtype of this disease involves the subcortical structures, including the nucleus of the brain stem, and presents as early ataxia and late dementia. The manifestations of the MV2 subtype, which has clear cerebellar involvement are similar to those of VV2, with early ataxia that gradually progresses to dementia over time. The MM2 subtype is characterized by well-defined spongy changes in the thalamus and lower olives, and manifests as sleeplessness, agitated behavior, ataxia, and cognitive alterations. Dementia is a manifestation of both MM2 and VV1 subtypes. The MM2 subtype is associated with pathological changes in all cortical layers, while the VV1 subtype is characterized by abnormalities of the cortical area and striatum. Although these categorizations are useful, they do not fully represent the broad spectrum of these illnesses, and as many as 35% of patients present with a mixed phenotype [6].

The most common symptom is cognitive dysfunction, followed by cerebellar, constitutional, and behavioral changes in approximately 25% of cases [7]. About one-third of patients with sporadic Creutzfeldt–Jakob Disease show prodromal signs, including asthenia, headache, malaise, vertigo, changes in sleep or eating patterns, and weight loss [7,8]. Approximately one-fifth of patients initially present with behavioral changes, which develop later in about 50% of patients during the course of the disease. Higher cortical dysfunctions including aphasia, apraxia, negligence, and acalculia, among others, are early disease indicators in about 5% of patients [7]. Vision or oculomotor impairment occurs early in approximately 10% of cases, and develop during the course of the disease in approximately 35% of cases. Additionally, about 7% of patients with sporadic Jakob–Creutzfeldt disease present with sensory symptoms [7]. Creutzfeldt–Jakob disease mimics several other neurological or psychiatric diseases, which often results in incorrect diagnoses [9].

### 2.2. Sporadic Fatal Insomnia

Sporadic fatal insomnia (FI) is a rapidly progressive neurodegenerative disease characterized by progressive insomnia that is followed by dysautonomia, stupor, and death [10]. The clinical manifestations include sleep abnormalities, psychiatric disorders, gait problems, and mobility disturbances. Pathological findings are observed in the thalamus and lower olives [11], with elevation of type 2 PrP^sc^ commonly identified in patients with MM homozygosity [10,12].

### 2.3. Variably Protease-Sensitive Prionopathy

Patients with MM homozygosity demonstrate significant Parkinsonism and myoclonus, with no psychiatric or cognitive involvement. In contrast, patients with VM and VV genotypes have significantly higher levels of psychiatric dysfunction and dementia than those without Parkinsonism and myoclonus. Approximately half of all patients with the three polymorphisms have been observed to have ataxia. CSF 14-3-3 protein, EEG, and MRI examinations are generally not useful for diagnosis [13]. Spongiform and glio-changes are observed diffusely in the cerebral cortex, basal ganglia, thalamus, and cerebellum [14,15].

## 3. Genetic Human Prion Diseases

### 3.1. Familial Creutzfeldt–Jakob Disease

Most patients with genetic prion disorders have unknown family history. Familial Jakob–Creutzfeldt disorder generally presents as rapidly progressive dementia and ataxia accompanied by motor abnormalities. Disease onset is observed between 30 and 60 years of age. Many cases of familial Creutzfeldt–Jakob disease are caused by the E200K variant, and are typically characterized by rapidly progressive dementia, myoclonus, and ataxia. MRI typically reveals symmetric striatal T2w/DWI hyper-intensities, usually with reduced cortical ribboning [16]. EEG patterns may vary in the family of variants responsible for Creutzfeldt–Jakob disease; however, a late periodic sharp wave complex is typical. CSF markers, including 14-3-3 protein, NSE, and t-tau, may be elevated, albeit with a lower frequency than that observed in sporadic Creutzfeldt–Jakob disease. A previous study has reported that real-time quaking-induced conversion (RT-QuIC) from CSF has a higher sensitivity than 14-3-3 protein or t-tau for the diagnosis of familial Jakob–Creutzfeldt [17].

### 3.2. Gerstmann–Straussler–Scheinker Syndrome

This disorder has near-total penetration and is characterized by tremors, cerebellar ataxia, speech, and swallowing dysfunction, pyramidal signs, Parkinsonism, sensory dysesthesia, and cognitive symptoms. Disease onset may occur any time between 20 and 80 years of age, and the duration can vary from a few months to more than 10 years. The spectrum of onset, duration, and clinical manifestations may be narrower for specific variants. Codon 129 polymorphisms may also contribute to disease manifestation, with individuals who are homozygous for the MM genotype manifesting earlier onset of the disorder than those with an MV genotype at the same locus. This is also the case for the Pro102Leu mutation. In contrast, carriers of apolipoprotein E variants present with late onset of symptoms [18,19].

Gerstmann–Sträussler–Scheinker syndrome is a gradually progressive ataxic or motoric (e.g., Parkinsonian) disease with late-onset dementia. Approximately 10 *PRNP* variants are known to be associated with this syndrome, including P102L, P105L, P105T, A117V, Q145X, F198S, Q217R, and several OPRI [5]. The median age of onset is often 50–60 years of age, and ranges between 20 and 70 years of age, although with large variability commonly seen, even within families.

### 3.3. Familial Fatal Insomnia

Patients with this disorder initially report hypersomnia due to mood and psychiatric changes, which are related to abnormal nocturnal sleep patterns in the early stages of the disorder [20,21]. Onset usually occurs at the end of the fourth decade, and subjects typically experience a severely progressive inability to sleep for a couple of months, followed by dysautonomia, including hyperhidrosis, tachycardia, and hyperpyrexia. Cognitive and motor manifestations usually manifest later on in the disease. In more progressed cases, polysomnography demonstrates a reduction in typical sleep transients, total sleep time, realization of dreams, and disorganization of sleep cycles [22], finally leading to protracted periods of stupor. Autonomic dysfunction with hypertension, fever, palpitation on movement and gait disorders have also been observed [23], along with an increase in total metabolic demand with cachexia. Although MRI results are non-specific and show diffuse atrophy, positron emission tomography (PET) imaging reveals significant and moderate hypo-metabolism in the thalamus and corpus callosum, respectively [24]. Neuronal loss and glio-changes are prominently seen in the anterior ventral and mediodorsal thalamic nuclei, as well as in the inferior olives. CSF 14-3-3 protein has low-sensitivity for the diagnosis of this syndrome, and spongiform changes are known to occur very late during disease progression [25]. Even though a genetic test is essential for the definitive diagnosis of this disease, various schemes have been proposed to aid disease confirmation. As suggested by Krasnianski [26], these algorithms focus on clinical manifestations and polysomnography results. However, the diagnosis can be complicated by the absence of a family history due to the low-sensitivity of available confirmatory tests and atypical clinical signs. In brief, patients with this disease do not meet the classical criteria for Creutzfeldt–Jakob disease (CJD), and consequently prion disease is often not suspected.

### 3.4. Other PRNP Mutations

Truncating variants lead to diseases that have very unusual clinicopathological presentations, as dementia progresses over time, and is often similar to Alzheimer’s disease, frontotemporal dementia, and other neurodegenerative diseases with amyloid prion angiopathy and tauopathy [27].

## 4. Acquired Human Prion Diseases

### 4.1. Kuru

Spongiform encephalopathy has a typical duration of one year and is characterized by progressive ataxia, dysarthria, dysphagia, tremors, and motor dysfunction. Patients with dementia have fewer cognitive symptoms as compared to those with other prion diseases. This disease was caused by ritualistic endocannibalism, and women and children were more likely to be affected since they were also more likely to consume brain tissue [28].

### 4.2. Iatrogenic Creutzfeldt–Jakob Disease (iaCJD)

A few cases of Creutzfeldt–Jakob Disease (CJD) contracted the disease post transfusion with contaminated blood [29]. Contaminations could also be mediated by dura matter grafts and intracranial surgical devices. The clinical presentation of this disease is similar to that of sCJD, with typical symptoms including ataxia, rapidly progressing dementia, and myoclonus. Clinical manifestations related to growth hormone infusion tend to affect the cerebellum, with significant ataxia and cognitive dysfunction developing later in the course of the disease [30]. iaCJD is more likely to occur in youth [31], and, as observed for other prion disorders, codon 129 polymorphisms seem to affect susceptibility to, and incubation time of, the disease [32].

The clinical phenotype and MRI results for iatrogenic Creutzfeldt–Jakob disease are associated with dura matter overlap, as seen in sporadic Creutzfeldt–Jakob disease [33].

### 4.3. Variant Creutzfeldt–Jakob Disease (vCJD)

Manifestations of variant Creutzfeldt–Jakob disease often begin with a psychiatric prodrome, at least six months before the onset of neurologic symptoms, which include dysesthesia, cognitive dysfunction, cerebellar dysfunction, dystonia, myoclonus, and chorea. The median age of onset is 27 years (range, 10–70 years) for this disease, earlier than that of sporadic Creutzfeldt–Jakob disease, while the median disease duration of vCJD is typically 15 months [34]. Many patients with this disease are homozygous for methionine at codon 129 in *PRNP*, which indicates the possible role of codon 129 heterozygosity in susceptibility [34]. However, the MV129 codon was also seen in patients with variant Creutzfeldt–Jakob disease. Unlike other prion diseases, the PrP^Sc^ in this disease are found not only in the central nervous system (CNS) but also in the lymphatic system, possibly due to acquisition via oral or blood routes [27,34].

vCJD initially manifests as psychiatric symptoms that progress to ataxia, as well as movement and cognitive dysfunction within a year [34]. EEG findings and CSF 14-3-3 lack sufficient sensitivity to confirm diagnosis [35], and CSF RT-QuIC is often negative. MRI signal intensity in the pulvinar area of the thalamus is the most sensitive indicator (pulvinar sign) of infection, and is seen in as many as 90% of cases [36]. Although a definite diagnosis requires brain biopsy, abnormal prion proteins can be detected in lymphatic tissue, thus rendering tonsillar biopsy as the preferred choice of proof of infection [37].

## 5. Host Immune Reaction against Prion Diseases

The host immune response to intracellular prion infection can have detrimental effects. These responses may be minimal or pro-inflammatory [38,39], involving several cytokines, including TNF-alpha, interleukin-1, and interleukin-6 [40,41,42].

Prions enter the digestive tract following their consumption. However, as they are resistant to the acidic gastric milieu, only minimal protection against prion infection is achieved at this stage. Previous studies have demonstrated the ability of prions to pass through the stomach and enter the intestine, where they accumulate in Peyer’s patches [43]. Notably, the number of Peyer’s patches is positively related to prion infectivity, and, thus, these structures play a critical role in the pathogenesis of prion disease.

M cells lie scattered among typical enterocytes in the intestine, and facilitate antigen uptake from the intestinal lumen to mediate immunosurveillance. However, certain pathogens, including prions, hijack these cells to cause infections. Previous studies have demonstrated efficient transcytosis of prion pathogens via M-cells. Further, an oral challenge revealed that prion proteins enter M cells in Peyer’s patches to infect hosts, and that the depletion of M cells in an animal model reduced the rate of infection by prion pathogens [43,44].

Following passage through the follicle-associated epithelium of the Peyer’s patches, prions spread via a possible cell-mediated mechanism. Macrophages that engulf prion proteins may play minor roles in their transmission and spread [45,46,47,48]. More importantly, dendritic cells from gut-associated lymphoid tissue, such as Peyer’s patches, transcytose these pathogens for antigen presentation to lymphocytes. Prions, in turn, exploit these mechanisms for intercellular transmissions.

Follicular dendritic cells, also a type of antigen-presenting cell, play a critical role in prion transmission [49]. Previous findings have demonstrated that prion proteins can accumulate in follicular dendritic cells [50,51,52,53,54,55,56,57,58], and mice with depleted follicular dendritic cells experience fewer intracerebral prion infections. These cells function as primary antigen-presenting cells that stimulate follicular helper T cells to produce interleukin-21 for B-cell antibody class switching in response to foreign antigens. Follicular dendritic cells usually express PrP^c^, and consequently are primary targets for prions, which hijack them to aid their own transmission. Additionally, mice treated with the lymphotoxin-β receptor antibodies that kill follicular dendritic cells avoid prion splenic accumulation and experience slower prion neuro-invasion [59]. A different study, in which mice were treated with an inhibitor of the tumor necrosis factor receptor, reported similar observations on the prevention of prion infection. Additionally, mice lacking lymphotoxin-α and lymphotoxin-β, which are crucial for follicular dendritic cell functioning, experience fewer prion intraperitoneal infections [59,60,61]. These findings support the notion that follicular dendritic cells are vital for prion infectivity. Further, they play critical roles in the peripheral retention of prion pathogens within lymphoid tissues, and in the replication of lymphotropic prion strains. Chronic lymphocytic inflammation with follicular dendritic cell-dominant lymphoid follicles within affected organs enable ectopic prion protein replication, further supporting the possibility of a key role for these cells in prion pathogenesis.

After accumulation and replication in secondary lymphoid organs, such as follicular dendritic cells containing lymphoid follicles, prions disperse to the central nervous system. Animal models have demonstrated that this dispersal occurs through the autonomic nervous system. Previous studies have revealed that sympathectomy prevents or delays prion pathogenesis [62,63,64,65], and, in contrast, sympathetic hyperinnervation in the secondary lymphoid organs of transgenic mice facilitates prion pathogenesis and nervous system invasion. Prion proteins are, therefore, believed to be transmitted via the sympathetic nerves to the spinal cord and brain.

On reaching the brain, prions progressively aggregate in the CNS causing fatal synaptic spongiform encephalopathies, and neuronal losses with neuroinflammation. Prion-mediated neuroinflammation may vary from aggressive to occasionally minimal. This process typically involves the activation of astrocytes and microglia, which is a prominent feature in patients with prion diseases [66,67,68,69,70,71]. Microglia function to clear apoptotic neurons subsequent to prion accumulation and infection. However, they typically fail to efficiently degrade the prion pathogens themselves [69]. Microglia is a subtype of macrophage located in brain tissues. Prion proteins also enable the transformation of macrophages from M1-type macrophages to M2-type macrophages [72]. Further, cytokines released by microglia augment the pathogenesis of prion infections.

**Figure 1:**
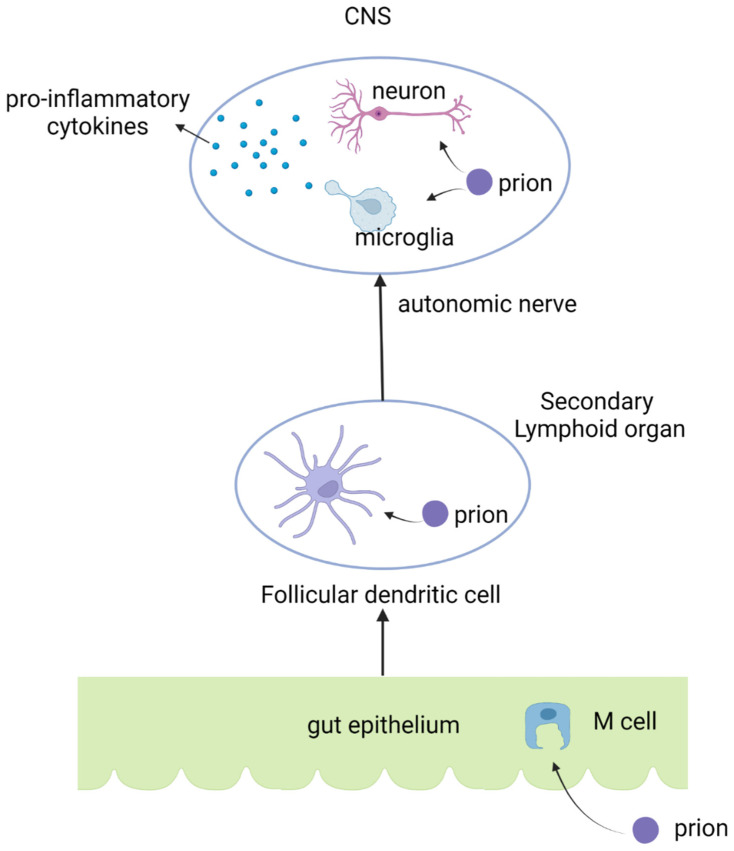
The infection route of prion pathogens and the induction of pro-inflammatory cytokines after prion infections.

Prion infections trigger NF-κB activation and the secretion of pro-inflammatory cytokines, including interleukin-1α, interleukin-1β, TNFα, and interleukin-6 [40,45], as has been observed in patients with prion diseases and in experimental mouse models. Additionally, the regulatory cytokine TGFβ is induced in mice after prion infection, which, in conjunction with interleukin-6, plays a key role in the TH17 immunological pathway. This indicates the possible induction of TH17 immunity subsequent to prion infection. Mice with depleted interleukin-1 receptors have also been observed to have significantly prolonged incubation periods for prion infection. The whole infectious process of prion pathogen infection is shown in Figure 1.

## 6. Protective Immunity against Prion Diseases

The THαβ immune response appears to be the protective host immunological pathway that targets prion diseases. Previous studies have found that type 1 interferons and interleukin-10 are protective against prion infections [73,74], and play crucial roles in the antiviral TGF-β immunological pathway. Interleukin-10 knockout mice have been shown to have shortened incubation periods for prion infection [75], and type 1 IFN administration protects animals from the same infection [73]. The key players in THαβ immunity include NK cells, CD8 T cells, IL-10-producing CD4 T cells, and IgG1 B cells [76]. The effector mechanisms of the THαβ immunological pathway are antibody-dependent cellular cytotoxicity (ADCC) executed by NK cells and MHC I-TCR-mediated cell cytotoxicity implemented via CD8 T cells [77]. Consequent to these processes, all intracellular protein and nucleic acid content is degraded via cellular apoptosis, resulting in stoppage of viral infectivity.

**Figure 2:**
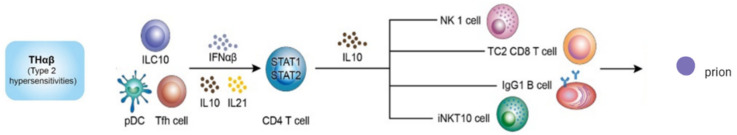
The protective THαβ immunological pathway against prion infection.

Several lines of evidence for the role of THαβ immunity in protection against prion diseases exist. Interferon regulatory factor 3 (IRF3) knockout mice show accelerated pathogenesis of prion infections [78]. IRF3, a MyD88-independent Toll-like signaling pathway mediator, functions downstream of the Toll-like receptor 3 (TLR3). Additionally, repeated TLR9 stimulation results in the initiation of protective immunity against prion infections [79,80,81]. TLR3 and TLR9 function as sensors of viral infection, and are responsible for initiating the THαβ immunological pathway, thereby potentially providing protective immunity against prion diseases. Furthermore, interleukin-10 knockout mice are more susceptible to prion diseases, post intraperitoneal or intracerebral inoculation with prion pathogens. Since interleukin-10 is the key mediator of THαβ immunity, the pathway is thought to be protective against prion infections. Type 1 IFN treatment has also been demonstrated to be protective in mice against prion infections. These molecules are the first host cytokines produced against viral infections. Moreover, CXCR3 knockout mice have been shown to accumulate prion pathogens with prolonged incubation periods in a prion infection challenge. The levels of CXCL9 and CXCL10, the ligands of CXCR3, as well as the CX3CR1–CX3CL1 axis, are known to change during prion infections [82]. CX3CR3 is a key chemokine receptor responsible for THαβ immunity, and the CX3CR1–CX3CL1 axis plays a critical role in antiviral immune responses. Host antiviral immune effects include the extermination of infected cells via the action of CD8 + T cells or NK cells [83]. Although intracellular bacterial and protozoan pathogens are killed by macrophages as part of TH1 immunity, intracellular prions digested by macrophages are not completely destroyed. Macrophages typically destroy intracellular pathogens via the action of lysozymes or the generation of free radicals subsequent to iNOS activation. This effect is exerted via the degradation of the bacterial cell wall by lysozymes, and lipid peroxidation of cellular membranes by free radicals. Proteins, however, are not highly susceptible to attack by free radicals, and prions in fact activate macrophages, such as microglia, to cause immune pathogenesis. Induction of antiviral THαβ immunity that results in apoptosis of prion-infected cells, accompanied by DNA fragmentation and protein degradation via the action of caspases, is critical in the defense against prion infections. Thus, apoptosis triggered by CTL or NK cells is the only successful immune response that degrades prion pathogens by utilizing the protein degradation machinery and consequently preventing further infection and transmission. Additionally, TH17 immunity plays a minor role in prion infections. TH17 immunity is known to use pro-inflammatory cytokines, including TNFα and IL-1, to activate neutrophils that, in turn, digest extracellular bacteria or fungi. Prions, not being extracellular pathogens, are not destroyed by neutrophils. However, they trigger TH17 immunity to mislead the host immune response and consequently prevent prion clearance by preventing appropriate functioning of THαβ immunity. Collectively, the above-mentioned findings suggest that host antiviral immunity is largely protective against prion pathogens. The protective immunity against prion infection is shown in Figure 2.

## 7. Conclusions

Although prion infections trigger pro-inflammatory cytokines that facilitate TH17 immunity, antiviral THαβ immunity provides protection against these pathogens. Currently, there are no effective medications for the treatment of prion infections. However, key mediators of THαβ immunity, including type 1 interferons, interleukin-10, and TLR3/TLR9 stimulators, can be exploited to initiate the host immune response against prion infections. This will contribute significantly to the development of strategies for the management of prion diseases.

## Data Availability

Not applicable.
